# Pathophysiological mechanisms of post-exertional malaise: an integrative analysis based on the metabolism-immune-neuro interaction model

**DOI:** 10.3389/fimmu.2026.1774310

**Published:** 2026-04-13

**Authors:** Hongjiao Jin, Yi An, Jingwei Huang, Tingting Luo, Xi Wu

**Affiliations:** 1 Acupuncture and Tuina School, Chengdu University of Traditional Chinese Medicine, Chengdu, Sichuan, China; 2 Department of Child Rehabilitation, The First People’s Hospital of Zunyi (The Third Affiliated Hospital of Zunyi Medical University), Zunyi, Guizhou, China

**Keywords:** chronic fatigue syndrome, energy metabolism, immunity, inflammation, mitochondrial dysfunction, neuroinflammation, Post COVID-19 condition, post-exertional malaise

## Abstract

Post-exertional malaise (PEM) is a common core symptom in various chronic debilitating conditions, such as Post COVID-19 Condition (PCC, also known as Long COVID) and Chronic Fatigue Syndrome (CFS). It is characterized by the delayed and persistent exacerbation of symptoms following even mild physical or cognitive activities. This review presents a systematic review of the pathophysiological mechanisms involved in PEM, proposing a dynamic framework of multi-system interactions that may lead to homeostatic imbalance. The etiology of PEM is multifactorial, potentially involving factors such as the persistent presence of pathogens, exposure to environmental toxins, and genetic predisposition. Collectively, these factors may establish a vulnerable baseline that heightens the body’s physiological response to stressors, such as exercise, potentially triggering a pathological reaction. First, mitochondrial dysfunction and metabolic abnormalities may act as potential initiating factors in PEM, manifesting as impaired ATP synthesis, overproduction of reactive oxygen species (ROS), and the accumulation of metabolic byproducts. It is crucial to emphasize that exercise itself induces a ‘toxic excitatory effect,’ whereby healthy individuals enhance mitochondrial function and antioxidant defenses through physical activity. However, in individuals predisposed to PEM, due to underlying pathological conditions (e.g., sequelae of viral infections), this adaptive process is disrupted, preventing effective restoration of mitochondrial homeostasis and may initiate a potential vicious cycle of dysfunction. Second, ROS and mitochondrial DNA (mtDNA), as damage-associated molecular patterns (DAMPs), along with pathogen-associated molecular patterns (PAMPs), may activate the NLRP3 inflammasome and induce the release of pro-inflammatory cytokines such as IL-1β, IL-6, and TNF-α, potentially transforming localized metabolic stress into a systemic inflammatory response. Subsequently, peripheral inflammation may be transmitted to the central nervous system through disruption of the blood-brain barrier and vagal nerve pathways, activating glial cells and initiating neuroinflammation. This process may ultimately affect the brain’s interoceptive network, particularly the insular cortex, resulting in altered perception and processing of signals related to fatigue and pain. Furthermore, mitochondrial dysfunction in neurons may contribute to central energy depletion, which may impair synaptic plasticity and induce cognitive deficits and brain fatigue. Ultimately, this review proposes that PEM may arise from a complex interplay among mitochondrial dysfunction, immune activation, and neuroinflammation, which together form a self-perpetuating loop of “energy exhaustion - inflammation amplification,” potentially contributing to the chronic and multi-system nature of PEM symptoms. The integrated “metabolism-immune-neuro” interaction model presented in this article may provide a potential comprehensive framework for understanding PEM and highlights the need for a multi-target, collaborative intervention approach that may help disrupt the pathological cycle.

## 1 Introduction

Post-exertional malaise (PEM) is characterized by a significant worsening of symptoms, such as fatigue and cognitive dysfunction, following mild physical, cognitive, or emotional exertion that the patient previously tolerated well. This exacerbation may occur immediately after exertion or may be delayed for several hours to days (1, 2).PEM not only exacerbates the symptom burden in patients (3–5), but also may lead to a loss of work capacity in some individuals (6, 7), and may even progress to a bedridden state (8), significantly reducing quality of life (9). It also severely disrupts psychosocial functioning, presenting as social difficulties, depression, emotional distress, etc. (3), sparking significant academic interest in the condition. PEM is commonly observed in a range of post-viral illnesses and chronic debilitating conditions, such as Post COVID-19 Condition (PCC, also known as Long COVID) (10), Chronic Fatigue Syndrome (CFS) (11), Q-fever Fatigue Syndrome (QFS) (12), Fibromyalgia (FM) (13), Gulf War Illness (GWI) (14), and Cancer-related Fatigue (CRF) (15), with particular prominence in PCC (10), and CFS (16).

**Figure 1:**
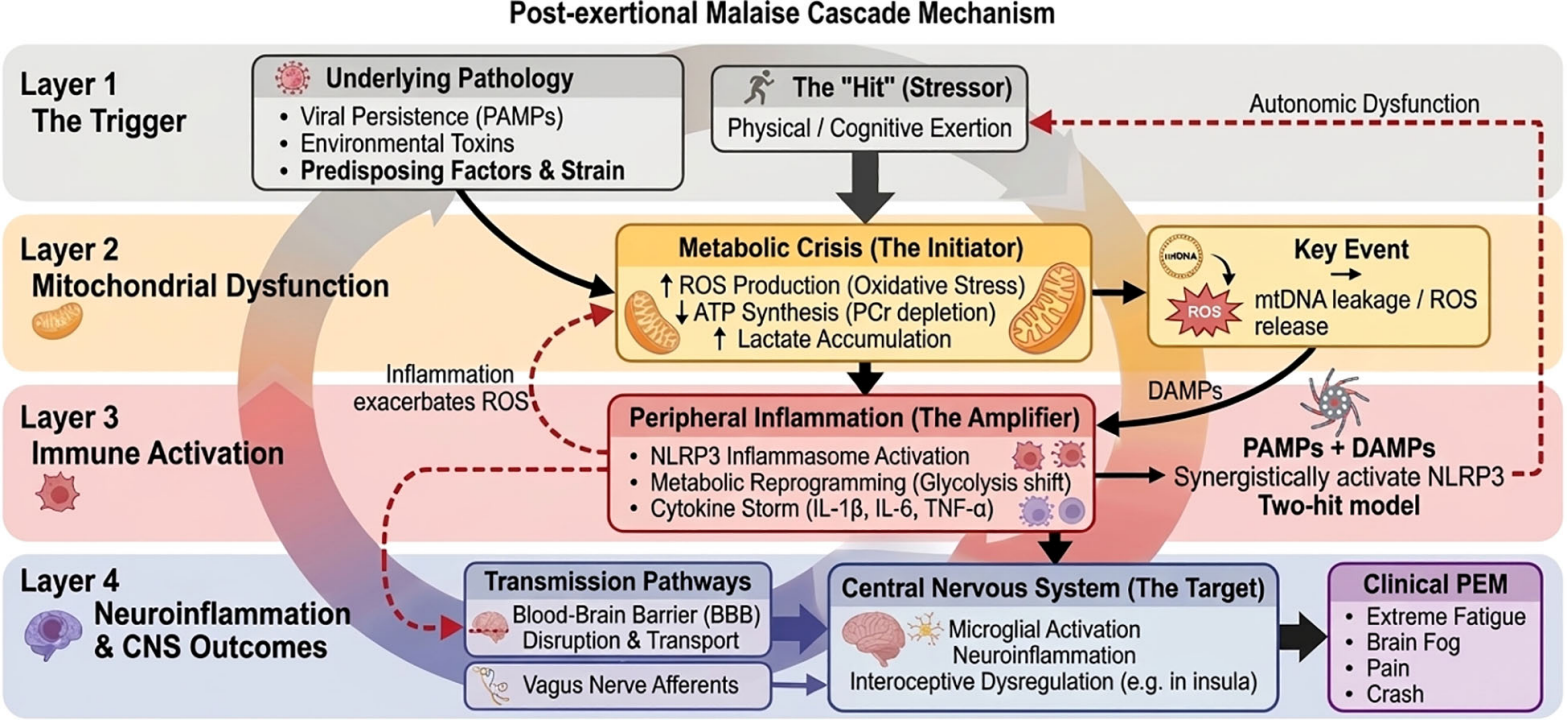
The Two-Hit Model and Provocation Framework for Post-Exertional Malaise (PEM).

A 2025 cross-sectional study in China, involving 12,789 individuals, reported that the prevalence of PCC within one year was 7.8%, with PEM affecting 36.9% of participants, making it the second most common symptom after fatigue (60.1%) (17). Longitudinal studies have further confirmed the marked long-term persistence of PEM, with approximately 35.6% of patients continuing to experience exercise intolerance two years after infection (18). This is closely associated with poorer overall health outcomes, including higher fatigue scores (18). Notably, a substantial proportion of PCC patients with PEM meet the diagnostic criteria for ME/CFS based on their clinical characteristics. Although the precise pathophysiology of PEM in PCC remains unclear, previous research on ME/CFS, along with current pathological evidence from PCC, suggests that it is not the result of a single system dysfunction, but rather involves widespread pathophysiological alterations. The prevalent view suggests that mitochondrial dysfunction, immune-inflammatory dysregulation, and nervous system injury are likely core mechanisms contributing to PEM (19–21). To investigate these mechanisms in greater depth, researchers often use provocation models, where patients undergo standardized physical exertion (e.g., cardiopulmonary exercise tests) or cognitive stress tests to monitor the dynamic pathophysiological worsening induced by exertional stimuli (22, 23). These provoked physiological abnormalities typically provide a more accurate reflection of the nature of PEM than those observed in a resting state (**Figure 1****).**

The key to understanding PEM lies in elucidating why exercise, which is generally protective in healthy individuals, induces pathological exacerbation in patients with PEM (24). In healthy individuals, exercise exerts a ‘toxic excitatory effect,’ where moderate oxidative stress enhances the body’s antioxidant defenses and promotes mitochondrial biogenesis. In contrast, PEM patients lose this adaptive capacity. A latent pathological background may be a key contributor to this dysfunction, which includes, but is not limited to: 1) the persistent presence of pathogens or their components (25); 2) exposure to environmental toxins; 3) complex disruptions in energy-sensing mechanisms, such as insulin resistance, abnormal leptin levels, and neuroendocrine dysregulation, that may impair the body’s ability to sense and allocate metabolic resources (26); and 4) autoimmune responses. Collectively, these factors may ‘pre-activate’ the immune system and increase mitochondrial vulnerability, potentially rendering what would normally be a routine exercise into a trigger for an uncontrollable and amplified pathological cascade. Consequently, the onset of PEM is thought to follow the ‘Two-hit model’ (25, 27, 28). The first ‘hit’—such as infection or toxin exposure—may establish a state of susceptibility, with exercise acting as the second hit that ignites the potential pathological cycle. The crux of this distinction lies in a reversal of homeostatic regulation: in healthy individuals, metabolic stress enhances bodily functions through the ‘toxic excitatory effect,’ whereas in PEM patients, sustained immune activation may cause the same exercise stress to trigger inflammatory cascades and oxidative damage, potentially converting adaptive stress into pathological depletion. The first hit primes the immune system and impairs mitochondrial homeostasis, while the second hit (exercise) exacerbates metabolic stress that further activates immune-inflammatory pathways, forming a potential feedforward loop between metabolism and immunity.

Thus, our study aims to synthesize pathophysiological evidence of energy metabolism, immune inflammation, and neurovascular system abnormalities observed following physical or cognitive exertion. By integrating research findings from PCC and ME/CFS, we seek to construct a pathophysiological framework that may account for multi-system interactions, potentially providing a more comprehensive understanding of the onset and progression of PEM.

## 2 Mitochondrial dysfunction and energy metabolism abnormalities

Mitochondrial dysfunction and the subsequent abnormalities in energy metabolism are thought to be one of the core potential mechanisms underlying PEM. As the central hub of cellular energy production, mitochondrial damage not only may lead to a decline in ATP synthesis but also may disrupt immune activation and neuronal function through metabolic signaling. This cascade of events may play a crucial role in the development of PEM. In this section, we provide a comprehensive analysis of the underlying mechanisms from three perspectives: mitochondrial stress, insufficient energy supply, and metabolic disorders.

### 2.1 Mitochondrial stress and structural damage

Mitochondria, as the primary organelles responsible for cellular energy metabolism, are highly sensitive to exercise-induced stress. This sensitivity is evident in multiple aspects, including mitochondrial structure, function, and metabolism (29–31).”In a healthy physiological state, exercise-induced mitochondrial stress elicits a phenomenon known as mitohormesis (mitochondrial hormesis) (32). This adaptive response is characterized by a transient, physiological increase in reactive oxygen species (ROS) that acts as a signaling mechanism to upregulate antioxidant enzymes and promote mitochondrial biogenesis, thereby enhancing the body’s resilience (33). However, in patients with PEM, this adaptive response is disrupted and may shift towards a pathological state. High-intensity or prolonged exercise may reduce mitochondrial membrane potential (ΔΨm) (34, 35), impair the activity of the electron transport chain (particularly Complex I) (36, 37), and promote electron leakage (38). These alterations may significantly increase the production of ROS (39), potentially triggering oxidative stress (40).

Studies indicate that patients with PCC or ME/CFS often exhibit an initial disruption in redox homeostasis. Due to the potential persistence of pathogen remnants or chronic low-grade inflammation, their antioxidant reserves are prematurely exhausted (41, 42). ROS have a dual role: on one hand, they serve as important signaling molecules in cellular signal transduction (43), while on the other hand, excessive ROS levels may oxidize mitochondrial DNA (mtDNA) (44, 45), membrane lipids, and mitochondrial proteins (46), leading to mitochondrial structural damage and potentially accelerating functional impairment (47, 48). This may initiate a potential vicious cycle where oxidative stress and mitochondrial damage mutually aggravate each other, causing further cellular harm (49). Studies indicate that a decrease in ΔΨm following exercise may lead to an imbalance between mitochondrial fusion and fission, accompanied by reduced ATP synthesis, and increased ROS production (30, 50, 51). Under this context, damaged mitochondria that are not effectively removed via mitophagy accumulate, potentially exacerbating oxidative stress and metabolic dysfunction (52, 53). Impaired mitophagy not only hampers mitochondrial quality control (54, 55), but also may trigger the release of pro-inflammatory factors and pro-apoptotic signals from dysfunctional mitochondria (56), thereby worsening cellular stress and potentially contributing to pathological changes, such as muscle atrophy (57). ROS can also activate the NF-κB and MAPK signaling pathways, which induce the expression of inflammation-related genes, thus providing a potential molecular basis for subsequent immune and neuronal responses (58, 59); these signaling pathways represent a key molecular link between mitochondrial oxidative stress and the subsequent activation of pro-inflammatory gene expression, connecting metabolic dysfunction to innate immune activation. Additionally, mitochondrial stress may disrupt Ca²^+^ homeostasis, activate the mitochondrial permeability transition pore (mPTP), and facilitates the efflux of mitochondrial matrix Ca²^+^, leading to mitochondrial swelling, outer membrane rupture, and subsequent dysfunction (60–62). This process releases damage-associated molecular patterns (DAMPs), which may activate immune responses through pattern recognition receptors (PRRs), potentially ultimately triggering systemic inflammation and stress responses (63–65). Thus, the ROS generated by the second hit of exercise no longer function as amplifying signals but instead exceed the damage threshold, potentially resulting in irreversible damage to mitochondrial DNA and membrane structures (66). In healthy individuals, these signals are transient and provoke adaptive responses, such as the upregulation of antioxidant enzymes (67). However, in patients with PEM, due to underlying pathological factors, such as persistent viral infections, mitochondrial autophagy and repair mechanisms are impaired, hindering the effective resolution of mitochondrial stress and initiating a potential pathological feedback loop (25).

### 2.2 Decline in ATP synthesis and insufficient energy supply

The traditional view asserts that the marked decline in ATP synthesis resulting from mitochondrial dysfunction is a potential primary contributor to the muscle weakness and fatigue observed in PEM (68, 69). Neurons, along with other high-energy-demand cells, are highly reliant on mitochondrial energy production, and inadequate ATP levels may compromise the efficiency of synaptic transmission (70), Furthermore, insufficient ATP activates a cellular stress response via the AMP-activated protein kinase (AMPK) signaling pathway (71, 72), suppressing non-essential metabolic processes (73), regulating mitochondrial biogenesis, and modulating autophagy (74) to conserve energy resources. Studies have shown that mitochondrial ATP production rate (MAPR) remains low during the post-exercise recovery period (75), accompanied by a temporary decrease in the efficiency of oxidative phosphorylation (OXPHOS) (76). Insufficient ATP synthesis directly impairs muscle contraction and may accelerate fatigue progression (77). In clinical studies, patients with ME/CFS show reduced ATP production following exercise, potentially resulting in persistent fatigue and delayed recovery (69). ATP insufficiency also adversely affects neuronal function by disrupting Ca²^+^ regulation, impairing synaptic activity and neurotransmitter synthesis (78–80)., and activating the AMPK-mTOR pathway to inhibit protein synthesis and cellular repair (81, 82).

However, the ‘energy deficit’ hypothesis fails to fully account for a key clinical feature of PEM: the significant decline in muscle strength (myasthenia) upon the initial contraction after sufficient rest, rather than solely following endurance activities (83). This suggests that, in addition to a reduction in the absolute amount of ATP, there may be upstream mechanisms influencing the immediate utilization of energy. Notably, the rate of post-exercise phosphocreatine (PCr) resynthesis—a direct marker of *in vivo* mitochondrial oxidative phosphorylation capacity—has been positively correlated with the recovery of muscle endurance following exercise (22, 84). In PEM patients, this delayed restoration of high-energy phosphate pools may indicate a fundamental deficit in mitochondrial respiratory function rather than a simple depletion of fuel reserves, and instead may point to more fundamental impairments in excitation-contraction coupling. Recent studies provide a new perspective on this, particularly the dysfunction of Na^+^/K^+^-ATPase (sodium-potassium pump) (83). Wirth and Steinacker (2026) comprehensively elucidated the central role of this enzyme in maintaining resting membrane potential and muscle excitability, noting that its dysfunction in PEM may arise from multiple factors, including diminished hormonal signaling, direct suppression by ROS, and inadequate ATP supply (83). A reduction in Na^+^/K^+^-ATPase activity directly results in membrane depolarization, which not only impairs the efficiency of action potential generation and propagation—potentially constituting a direct electrophysiological factor for myasthenia at rest—but also brings the membrane potential closer to threshold, thereby inducing muscle hyperexcitability (manifested as fasciculations and painful spasms). These involuntary excitations further exacerbate Na^+^ influx, increasing the burden on the already compromised Na^+^/K^+^-ATPase, creating a potential vicious cycle of membrane depolarization and ion pump failure (83). More importantly, the dysfunction of Na^+^/K^+^-ATPase leads to secondary mitochondrial damage through ion imbalances: intracellular Na^+^ accumulation promotes reverse operation of the sodium-calcium exchanger (NCX), resulting in intracellular calcium overload (85), which is a key potential trigger for mitochondrial structural damage and functional impairment (86). Thus, the ATP deficiency observed in the traditional view may (68, 69, 75), to a significant extent, be a downstream consequence of upstream ion pump failure and subsequent calcium overload. In summary, the myasthenia observed in PEM may not stem from ATP depletion per se, but rather from the dysfunction of the ion pumps themselves (87).

The Na^+^/K^+^-ATPase dysfunction and subsequent calcium overload not only impair energy utilization but also further promote mitochondrial ROS production and mtDNA release, which may serve as DAMPs to activate downstream immune pathways, forming a metabolic-immune cross-talk at the cellular level. Thus, the ‘energy deficit’ observed in PEM is not merely a result of isolated ATP depletion, but rather a multifaceted process centered around Na^+^/K^+^-ATPase dysfunction, involving the interaction between impaired energy utilization (failure to maintain membrane potential) and deficits in energy production (secondary mitochondrial damage). Within this framework, traditional concerns regarding ATP synthesis deficits (68, 69, 75), dysregulated post-exercise phosphocreatine (PCr) resynthesis kinetics (88, 89),metabolic stress via the AMPK pathway (71–74), and the direct impact of ATP insufficiency on neuromuscular function can all be integrated as downstream events stemming from this central mechanism (78–80). This conceptualization not only offers a more comprehensive potential explanation for the dual clinical features of immediate and delayed exacerbation of myasthenia but also provides a more robust potential molecular basis for linking subsequent immune activation with neuroinflammation.

### 2.3 Metabolic disorder and oxidative stress

Mitochondrial dysfunction may trigger widespread disturbances in energy metabolism through multiple pathways. First, regarding lactate metabolism, hypoxia and mitochondrial damage induce a shift toward glycolysis for compensatory ATP production, potentially leading to lactate accumulation (90, 91). Some studies suggest that exogenous lactate supplementation may mitigate hypoxia-induced mitochondrial dysfunction and cellular apoptosis (92). Second, mitochondrial damage disrupts the redox balance of NAD^+^/NADH (93). Impairment of the citric acid cycle leads to a decreased NAD^+^/NADH ratio (94, 95), and damage to mitochondrial Complex I exacerbates redox imbalance in tissues, such as the pancreas (96). Additionally, lipid peroxidation may represent another important pathological process resulting from mitochondrial dysfunction. ROS directly damage membrane lipids, triggering lipid peroxidation, while the ERRγ-GLS1 axis regulates the ability of GPX4 to clear lipid peroxides by modulating the GSH/GSSG ratio (97). Moreover, iron accumulation and impaired autophagy have been shown to contribute to lipid peroxidation in neurodegenerative diseases (98, 99). Proteomic studies also reveal significant dysregulation of energy metabolism pathways, including glycolysis and OXPHOS, following exercise (100, 101). These metabolic abnormalities further influence immune cell metabolism and neuronal function through signaling byproducts such as lactate and ROS, thereby potentially linking metabolism, immunity, and neurobiology in the pathogenesis of PEM (102). Experimental studies indicate elevated levels of lactate, pyruvate, and lipid peroxidation products in the blood of PEM patients (23, 103), which correlate positively with post-exertional fatigue. According to consensus guidelines for the assessment of oxidative stress biomarkers, the multi-parametric evidence of oxidative damage—particularly the lipid peroxidation products identified through advanced techniques such as mass spectrometry—may provide supportive evidence of potential redox imbalance and reduced antioxidant capacity in PEM patients (104).

**Figure 2:**
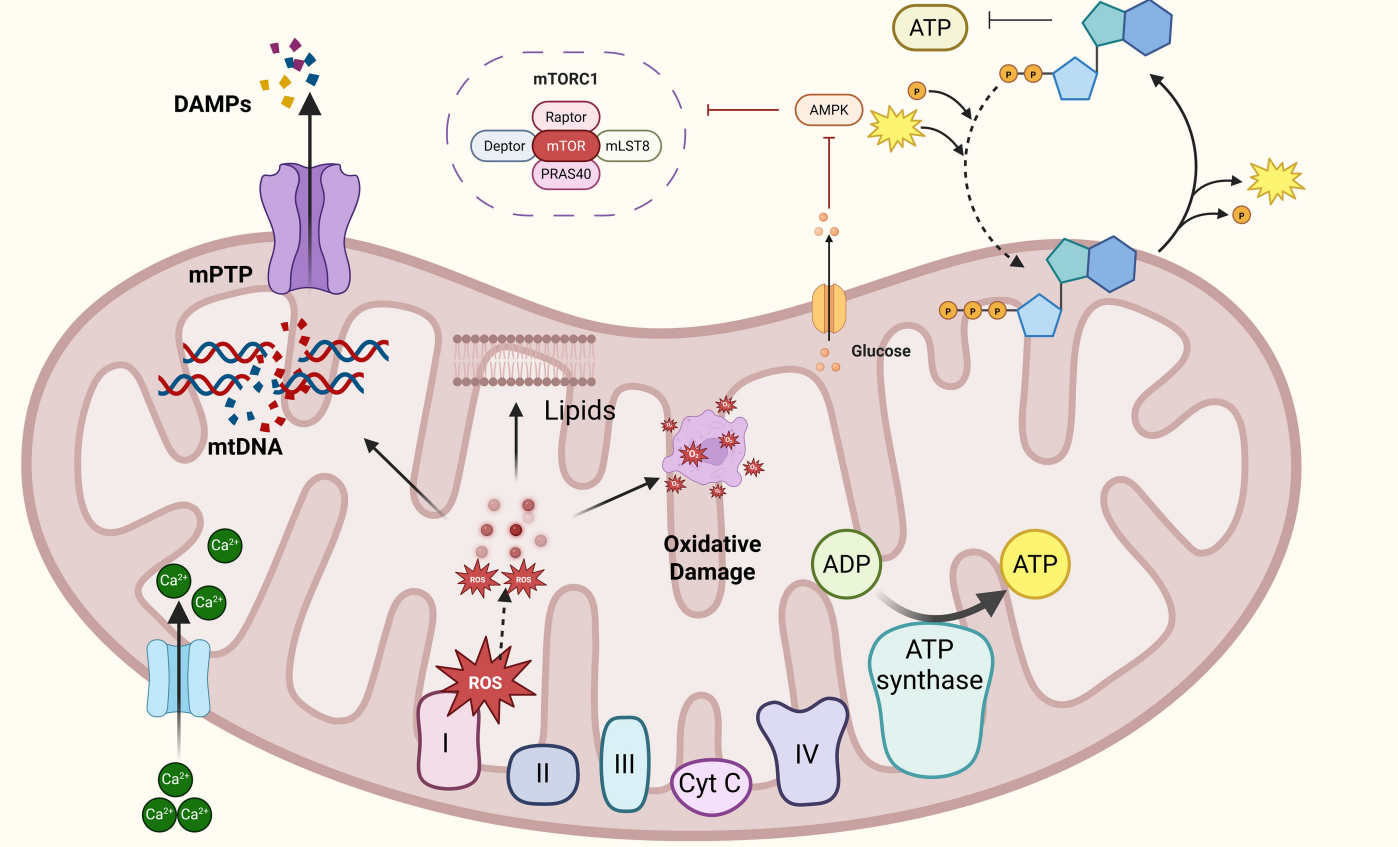
Mitochondrial Stress and Bioenergetic Failure as Initiating Factors in PEM.

Mitochondrial dysfunction-induced metabolic disorders (e.g., excessive ROS, lactate accumulation, mtDNA release) generate a series of damage-associated molecular patterns (DAMPs) that can be recognized by pattern recognition receptors (PRRs) on immune cells. For example, mtDNA can bind to TLR9, and ROS can activate the NLRP3 inflammasome priming step, which may be the core molecular mechanism by which metabolic stress initiates peripheral immune activation. These metabolic-derived DAMPs, together with potential pathogen-associated molecular patterns (PAMPs) in PCC, may synergistically enhance immune cell activation, forming a critical link between mitochondrial metabolic dysfunction and the subsequent inflammatory response. In summary, mitochondrial stress, reduced ATP production, and metabolic disorders may constitute the potential metabolic foundation of PEM. Mitochondrial dysfunction not only may cause local and systemic energy deficits but also connects immune activation and neuronal damage through signals such as ROS, Ca²^+^, and metabolic byproducts. This interaction may provide the potential foundation for the development and persistence of PEM symptoms, establishing a potential molecular and metabolic framework for the pathophysiology of PEM. Importantly, it is crucial to recognize that these changes represent fundamental physiological responses to exercise-induced stress. However, in PEM patients, due to a lack of effective negative feedback regulation (e.g., compromised antioxidant capacity and impaired mitochondrial autophagy), these responses fail to resolve post-exercise, thereby potentially progressing into a chronic pathological state (105) (**Figure 2**).

## 3 Immune activation and inflammatory response

The onset of PEM is closely linked not only to mitochondrial dysfunction and energy deficiency but also to the dysregulated activation of the immune system and inflammatory reactions. ROS and DAMPs generated by mitochondrial damage may trigger the activation of peripheral immune cells. These activated immune cells, via the bloodstream and neural pathways, may exert effects on the central nervous system, thereby potentially establishing a metabolic–immune-neuro interaction loop. Crucially, emerging evidence indicates that the persistence of viral reservoirs or residual pathogen-associated molecular patterns (PAMPs)—such as the circulating SARS-CoV-2 Spike protein detected months post-infection—may serve as potential primary initiating triggers for this chronic immune activation (106, 107). PAMPs deliver essential pro-inflammatory signals via Toll-like receptors (TLRs), thereby priming the immune system and increasing the likelihood that subsequent mitochondrial stress will escalate into a pathological inflammatory cascade (106). However, DAMPs alone are insufficient to explain the initiation of PEM, especially in post-infectious syndromes such as PCC, where PAMPs may play a crucial role (25, 27). This section discusses the role of immune activation in PEM, with a focus on three key areas: mitochondrial-driven inflammatory signaling, peripheral inflammatory manifestations, and immune-metabolic reprogramming.

### 3.1 Mitochondria-driven inflammatory signaling

Mitochondrial dysfunction may lead to the overproduction of ROS and the release of mtDNA into the cytoplasm (44). The abnormally released mtDNA and overproduced ROS are recognized by the immune system as DAMPs (108–110), which subsequently may activate the NLRP3 inflammasome (111, 112). The activated NLRP3 inflammasome processes pro-IL-1β and pro-IL-18 into their mature forms through caspase-1 (113, 114), promoting the secretion of these pro-inflammatory cytokines (115, 116). This cascade of events may ultimately trigger a systemic inflammatory response (117). This process exemplifies the two-hit model underlying the mechanisms of PEM. The first hit arises from the immune imprint left by PAMPs following the initial infection, which sensitizes immune cells to an elevated state (22). The second hit is represented by exercise-induced mitochondrial DAMPs (such as released mtDNA and ROS) (118). This synergistic interaction explains why PEM patients’ responses to exercise-induced stress far exceed normal thresholds and why mitochondrial dysfunction may be a consequence of sustained immune-metabolic reprogramming rather than an isolated, spontaneous event (22). Additionally, ROS can enhance the expression of pro-inflammatory factors such as TNF-α and IL-6 by activating key signaling pathways, including NF-κB and MAPK (119, 120). In overtraining animal models, significantly elevated serum levels of IL-1β, IL-6, and TNF-α have been observed, which correlate positively with mitochondrial ROS levels (121). This mechanism suggests that mitochondrial damage not only causes energy insufficiency but also may transform localized cellular stress into systemic inflammation via immune signaling, providing a potential precursor for subsequent neuroinflammation in PEM. PAMPs from persistent pathogen components and DAMPs from mitochondrial stress synergistically activate the NLRP3 inflammasome and NF-κB pathway, which are the core innate immune pathways mediating the conversion of metabolic stress into systemic inflammation. Following exercise or exertion, patients with ME/CFS exhibit elevated levels of pro-inflammatory cytokines in peripheral blood, accompanied by muscle fatigue, exhaustion, and cognitive impairments, indicating that mitochondrial-driven inflammatory signaling may be a potential key driver of PEM symptoms (69).

It is crucial to emphasize that, in many cases of PEM—particularly in post-infectious conditions such as PCC and ME/CFS—immune activation is not solely driven by endogenous DAMPs (107). The persistent presence of pathogens, such as viruses or bacteria, or their residual components (e.g., the spike protein of SARS-CoV-2), may function as pathogen-associated molecular patterns (PAMPs), continuously activating pattern recognition receptors (PRRs) such as Toll-like receptors (TLRs), thereby providing a potential foundational pro-inflammatory signal (25, 122). Studies have shown that PCC patients with symptoms lasting more than 30 days exhibit persistent SARS-CoV-2-specific T cells and heightened antibody affinity, strongly suggesting the continued presence of viral antigens (25). This PAMP-driven, low-grade chronic inflammation may significantly amplify the effects of subsequent exercise-induced DAMP signals and is central to understanding the ‘two-hit’ model of PEM. In other words, the first hit (infection) leaves a lasting immune imprint (driven by PAMPs), which primes the immune system, making the second hit (exercise) and its associated mitochondrial stress (driven by DAMPs) more likely to exceed the threshold, triggering a severe inflammatory cascade.

### 3.2 Central transmission of peripheral inflammation and the self-sustaining nature of neuroinflammation

Peripheral inflammation is characterized not only by elevated levels of pro-inflammatory cytokines (IL-6, TNF-α, IL-1β) in the circulatory (123, 124), but its pathological effects are also transmitted to the central nervous system via both humoral and neural pathways, thereby converting peripheral events into a potentially sustained central pathological state.

Peripheral inflammatory cytokines transmit signals to the central nervous system through two distinct pathways. 1) specific saturable transport systems facilitate the active transport of cytokines, such as IL-1β, IL-6, and TNF-α, across the BBB, allowing them to influence brain function even when the barrier is intact (125). 2) Under conditions of sustained systemic inflammation (as seen in PEM), high levels of circulating cytokines may degrade tight junction proteins (e.g., claudin-5 and occludin), leading to BBB disruption and increased paracellular permeability. Increased BBB permeability allows peripheral pro-inflammatory cytokines (e.g., IL-6, TNF-α) to infiltrate the central nervous system, where they directly activate microglia and astrocytes. Activated glial cells further release pro-inflammatory mediators and impair neuronal glucose metabolism, which may directly regulate the central fatigue circuits in the insular cortex and prefrontal cortex—key brain regions mediating the perception of fatigue and cognitive function. This disruption allows for the uncontrolled influx of peripheral immune cells and inflammatory mediators into the brain parenchyma, further exacerbating neuroinflammation (126).

The central issue lies in elucidating how transient events are transformed into long-term consequences. The transient elevation of peripheral cytokines, such as IL-6 induces sustained effects through the following mechanisms: 1) Activated microglia can sustain a pro-inflammatory phenotype over extended periods, thereby potentially becoming a continuous source of IL-1β and TNF-α within the central nervous system (127, 128). 2) Activation of endothelial cells and disruption of tight junction proteins within the neurovascular unit may result in persistent dysfunction of the BBB (129). 3) Inflammatory cytokines modulate synaptic plasticity and neuronal energy metabolism, potentially leading to cumulative synaptic damage and neuronal dysfunction (130–132). Furthermore, the interaction between mitochondrial dysfunction in peripheral immune cells and inflammation may further aggravate this pathological process (133). Collectively, these mechanisms may convert transient peripheral inflammation into a self-sustaining chronic inflammatory state within the central nervous system, ultimately manifesting as delayed fatigue, reduced attention, and cognitive impairment observed in PEM patients following physical or cognitive exertion (134, 135).

Clinical and experimental studies support this framework: Peripheral pro-inflammatory cytokines, such as IL-6, may disrupt the BBB and activate central inflammation, potentially leading to cognitive impairments (134). Conversely, inhibiting peripheral inflammation has been shown to significantly improve cognitive function following exercise (136, 137). Furthermore, vagus nerve stimulation has been demonstrated to mitigate central inflammation via the cholinergic anti-inflammatory pathway (138, 139), presenting a potential therapeutic target for alleviating cognitive symptoms in PEM (140). The CAP represents a key neural regulatory pathway by which peripheral immune signals are modulated to influence central neuroinflammation, and its dysfunction may further amplify the transmission of peripheral inflammatory signals to the central nervous system, exacerbating PEM-related cognitive and fatigue symptoms. Collectively, these findings suggest that the transmission of peripheral inflammation to the central nervous system, and its self-sustaining nature within the brain, may constitute a central potential mechanism linking acute exacerbations of PEM with long-term cognitive consequences.

### 3.3 Immune metabolic reprogramming

In an inflammatory state, immune cells undergo metabolic reprogramming, characterized primarily by enhanced glycolysis and diminished OXPHOS, to meet the heightened energy demands of the inflammatory response (141). However, the distinctiveness of PEM lies in the manner in which this reprogramming is triggered and its self-amplifying nature: it is not directly induced by pathogens but is instead initiated by a potential energy crisis resulting from mitochondrial dysfunction. Mitochondrial-derived ROS and mtDNA, acting as DAMPs, activate the NLRP3 inflammasome, prompting immune cells to shift toward glycolysis to maintain their function (42, 69). This ‘energy crisis-driven immune activation’ constitutes the fundamental distinction between PEM and the pathogen-driven inflammation observed in infectious diseases.

The metabolites generated during metabolic reprogramming, including lactate and ROS, further impair local mitochondrial function, potentially exacerbating the energy deficit (69) and establishing a potential characteristic metabolic-inflammatory feedback loop in PEM. The ensuing energy shortage compels immune cells to resort to less efficient energy production pathways, which in turn may lead to the generation of additional inflammatory mediators, thereby worsening mitochondrial dysfunction in peripheral and central cells (42). Research has demonstrated that, in the context of inflammation, enhanced glycolytic activity in macrophages and T cells promotes the secretion of IL-1β (142). Simultaneously, the accumulation of lactate can upregulate the expression of inflammation-associated genes through the activation of the HIF-1α signaling pathway (143–145).

More importantly, the outcomes of immune-metabolic reprogramming in PEM exhibit both tissue-specificity and temporal delays. Metabolites such as lactate not only may play a crucial role in immune modulation but also may contribute to central sensitization by altering the synaptic microenvironment (146), thereby potentially establishing a direct link between peripheral metabolic dysfunction and central nervous system inflammation. Furthermore, the initiation of this cascade is not instantaneous—spanning from mitochondrial damage and the release of damage-associated molecular patterns (DAMPs) to immune cell metabolic reprogramming and the subsequent production of inflammatory mediators. This temporal lag may provide a clear explanation for the delayed onset (ranging from hours to days post-activity) and the persistent nature (with the cycle remaining resistant to spontaneous resolution) of PEM symptoms (22, 23).

**Figure 3:**
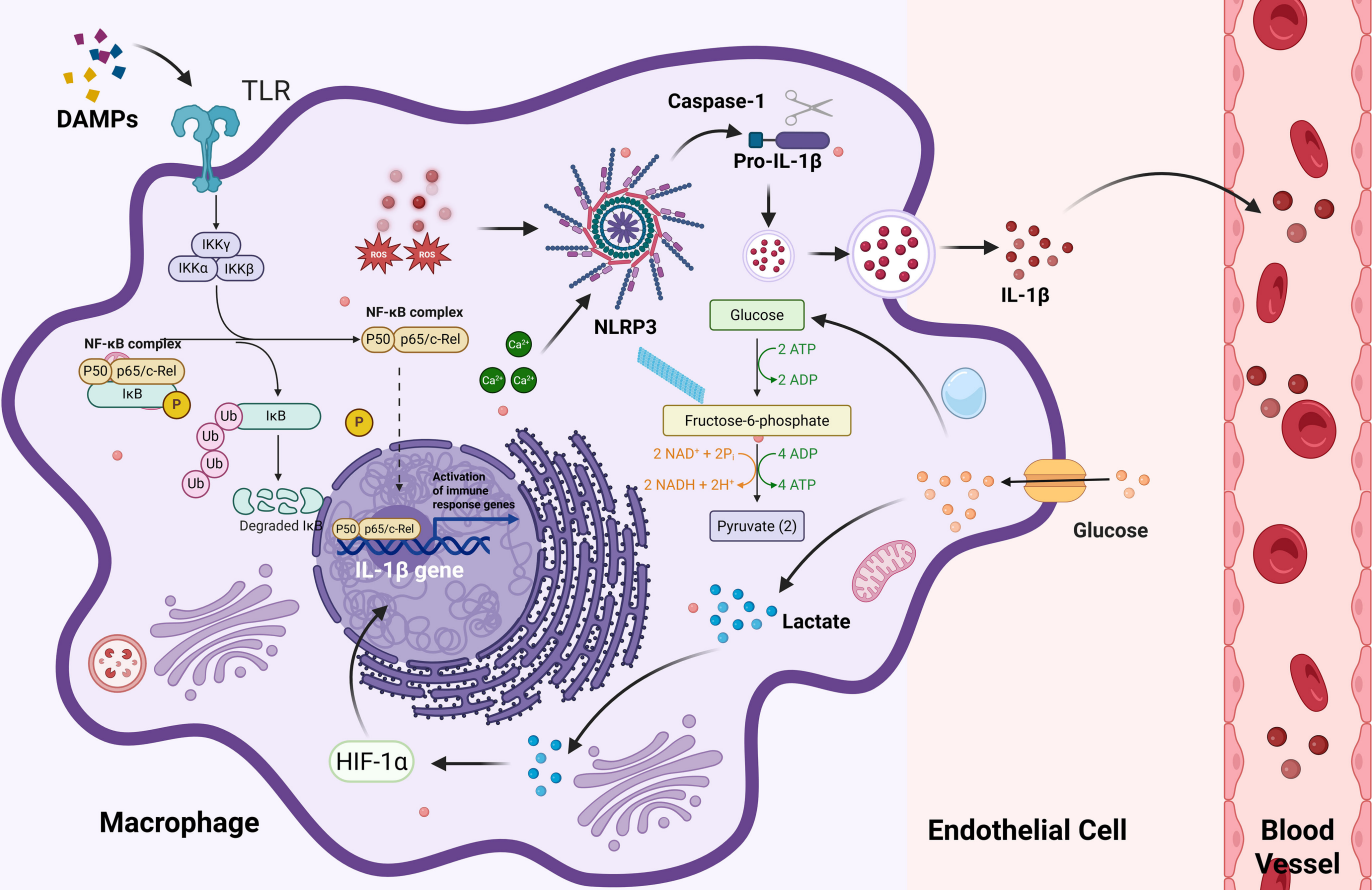
Immune Activation and Metabolic Reprogramming Driven by DAMPs and PAMPs.

Immune metabolic reprogramming in peripheral immune cells generates large amounts of lactate and pro-inflammatory cytokines, which can not only exacerbate peripheral mitochondrial dysfunction but also cross the compromised BBB to regulate glial cell activation and neuronal mitochondrial function in the central nervous system. For example, lactate can activate the HIF-1α pathway in microglia to enhance pro-inflammatory cytokine release, while IL-1β can inhibit neuronal PGC-1α expression to impair mitochondrial biogenesis, forming a potential link between peripheral immune metabolic reprogramming and central neuroinflammation and energy deficiency. Thus, immune activation in PEM should not be considered as a typical inflammatory response, but rather as a distinct pathological process instigated by an energy deficit. This process is marked by a metabolic-inflammatory positive feedback loop, exhibiting unique temporal dynamics and tissue specificity. This mechanism reveals the intricate, multi-layered interactions between metabolic dysfunction, immune activation, and neuroinflammatory damage, providing a potential molecular basis for the subsequent development of neuroinflammation (**Figure 3**).

## 4 Central energy deficiency and neuroinflammation

The hallmark symptoms of PEM, including cognitive decline, brain fatigue, and attention deficits, are closely associated with neuroinflammation within the central nervous system and intrinsic energy metabolism dysfunction in neurons. It is crucial to distinguish that the central energy deficit specifically refers to metabolic dysfunction within the central nervous system, impacting both neurons and glial cells (such as impaired glucose metabolism and mitochondrial dysfunction), rather than a deficiency in cerebral perfusion due to circulatory system dysfunction. The latter represents a central perfusion deficit or hemodynamic disorder, characterized by distinct mechanisms, although both conditions may coexist. Peripheral inflammatory signals and energy deficits may impact brain tissue through multiple pathways, activating microglial cells and astrocytes, disrupting neuronal metabolism and synaptic transmission, and thereby potentially contributing to the neurological phenotype of PEM. Recent research advancements indicate that the central nervous system symptoms associated with PEM, particularly fatigue and pain, can be conceptualized as ‘interoceptive dysregulation.’ Interoception refers to the process by which the central nervous system perceives, interprets, and integrates internal physiological signals from the body (147). The insular cortex serves as the primary hub of the interoceptive network, receiving signals from throughout the body and generating subjective sensations such as fatigue, pain, and breathlessness. In PEM, sustained peripheral immune-metabolic dysfunction transmits aberrant ‘body state’ signals to the brain, potentially resulting in interoceptive mismatch, which ultimately manifests as excessive fatigue and pain (147). This section discusses the role of neuroinflammation in PEM from three perspectives: the formation of central inflammation, neuronal dysfunction, and positive feedback mechanisms.

### 4.1 Central nervous system inflammation

Peripheral inflammatory signals enter the central nervous system through multiple pathways, including the BBB, vagus nerve, and humoral routes. In an inflammatory state, the levels of pro-inflammatory cytokines (such as IL-1β, TNF-α, and IL-6) are elevated, leading to a disruption in the structural integrity of the BBB and increasing its permeability. This allows peripheral immune cells and inflammatory factors to infiltrate the brain tissue (148, 149). Upon entering the brain, the inflammatory factors directly activate microglial cells, which release ROS, nitric oxide (NO), and additional pro-inflammatory cytokines, thus creating a local neuroinflammatory environment (150). Meanwhile, astrocytes are also activated in response to inflammation, influencing synaptic transmission efficiency by regulating both neuronal energy metabolism and clearance of glutamate (151). Additionally, the vagus nerve plays a crucial role in neuroimmune regulation. Its afferent fibers (AFVN) and the dorsal motor nucleus (DMN) regulate both peripheral and central inflammatory responses through the cholinergic anti-inflammatory pathway (CAP) (138, 152, 153).

Positron emission tomography (PET) studies provide direct *in vivo* evidence supporting the aforementioned processes. Research employing PET tracers targeting the mitochondrial translocator protein (TSPO), such as [¹^8^F]DPA-714, has demonstrated that some PCC patients continue to exhibit widespread elevated TSPO signals in the brain up to two years post-infection, with the intensity of this increase being most pronounced in individuals with persistent fatigue and cognitive symptoms (147) Animal studies have further substantiated that the sustained elevation of TSPO-PET signals (lasting at least 50 days) following SARS-CoV-2 infection arises from the co-activation of microglia, astrocytes, and endothelial cells, and that this neuroinflammatory state can endure long after viral clearance in the peripheral tissues (154). Systematic reviews have also highlighted similar neuroinflammatory evidence in ME/CFS patients, though significant individual variability exists, with some studies failing to detect notable differences in TSPO binding (155). Importantly, the most common cognitive complaint in PCC patients, ‘brain fog,’ closely correlates with the regions of neuroinflammation identified by TSPO-PET imaging, providing a potential objective neuroimaging basis for these subjective symptoms.

Animal studies have further corroborated this mechanism: the extent of microglial activation following exercise positively correlates with cognitive impairment scores, and inhibiting microglial activation significantly mitigates post-exercise cognitive dysfunction (156, 157). These findings suggest that central nervous inflammation may act as a direct potential driver of PEM symptom development, converting peripheral immune and metabolic dysfunction signals into neurofunctional impairments. The distinctive value of PET imaging lies in its capacity to provide non-invasive, visual evidence of neuroinflammation, thereby directly linking peripheral immune activation with central clinical manifestations, such as brain fog and cognitive decline, while also revealing the heterogeneity within patient populations.

### 4.2 Neuronal energy deficiency and cognitive decline

Mitochondrial dysfunction and insufficient energy supply in the central nervous system may lead to inadequate ATP production, which in turn may affect neuronal synaptic transmission efficiency and excitability, particularly impairing the function of high-energy-demand brain regions such as the prefrontal cortex, hippocampus, and basal ganglia (158, 159). This metabolism disorder not only may cause cognitive dysfunctions, such as decreased attention, memory impairment, and learning difficulties (160–162), but is also linked to mood fluctuations and disorders like depression (163, 164). ROS and pro-inflammatory cytokines further weaken neural network function by affecting synaptic plasticity and neurotransmitter release.

This central energy deficit has been visually confirmed through [¹^8^F]FDG PET imaging. Multiple studies have demonstrated widespread cerebral hypometabolism in PCC patients, particularly in regions such as the prefrontal cortex, cingulate cortex, insula, hippocampus, brainstem, and cerebellum (165). Notably, this hypometabolic pattern closely correlates cognitive function scores in these patients and cannot be fully attributed to local reductions in cerebral blood flow. Some studies have concurrently employed cerebral blood flow perfusion imaging, revealing that the hypometabolic regions extend beyond those with reduced perfusion, suggesting the presence of intrinsic mitochondrial dysfunction in neurons (165). In other words, even with adequate oxygen and glucose supply, neurons are unable to effectively utilize these substrates for oxidative phosphorylation. This finding aligns with the mitochondrial dysfunction discussed in Chapter 2: mitochondrial damage in peripheral skeletal muscle is reflected as regional glucose hypometabolism in the central nervous system. Neuronal mitochondrial dysfunction and central hypometabolism not only impair synaptic plasticity in cognitive-related brain regions but also further promote the release of neuronal DAMPs (e.g., mtDNA), which can activate local microglia in the central nervous system, forming a central energy-inflammation positive feedback loop.

Magnetic resonance spectroscopy (MRS) studies further substantiate the evidence of metabolic disturbances: in ME/CFS patients, brain lactate concentrations and the choline/creatine ratio are significantly elevated, indicating increased anaerobic glycolysis and altered membrane phospholipid metabolism, both of which are linked to neuroinflammation and mitochondrial oxidative stress (155). Animal studies have also shown that enhancing cerebral ATP supply or inhibiting inflammatory mediators can significantly improve post-exercise cognitive function (166, 167), further reinforcing the potential central role of neuronal energy deficits in the cognitive decline observed in PEM.

### 4.3 The vicious cycle of metabolic deficits and immune activation

Mitochondrial dysfunction may result in overproduction of ROS and the release of mtDNA, both of which serve as DAMPs, activating the NLRP3 inflammasome in both peripheral and central immune systems, thereby promoting the release of pro-inflammatory cytokines, such as IL-1β and IL-18 (168–170). At the same time, immune cells undergo metabolic reprogramming in response to inflammatory stimuli. While this process enables cells to rapidly acquire energy to support the inflammatory response in the short term, its inefficiency further exacerbates local energy depletion in peripheral and central tissues (171, 172). The absence of energy substrates not only limits the feedback regulatory capacity of immune cells but also further impairs the ability of affected tissues to maintain cellular homeostasis, creating a bidirectional amplification loop of “energy deficiency-exacerbated inflammation” (173). Microglia play a pivotal role in perpetuating this cycle. Upon stimulation by mitochondrial DNA (mtDNA), lipopolysaccharide-like signals, or peripheral inflammatory cytokines, microglia can adopt a ‘pro-inflammatory activated’ phenotype for prolonged periods in the central nervous system. The inflammatory signaling in microglia is primarily mediated through classical pathways such as TLR4/NF-κB and JAK/STAT, which are crucial for sustaining this activation (174, 175). TLR4-induced signaling cascades continuously activate the IKK complex, facilitating the translocation of NF-κB into the nucleus and driving the production of numerous inflammatory mediators (176). Simultaneously, IL-6 and IFN-γ further reinforce the pro-inflammatory state of microglia via the JAK/STAT axis, enabling the sustained expression of inflammatory cytokines even in the absence of persistent exogenous stimuli (175, 177). These inflammatory signals, in turn, inhibit neuronal mitochondrial biogenesis pathways by downregulating key regulatory factors such as PGC-1α and NRF1/2 (178), thereby impairing mitochondrial turnover and damage repair. This disruption may lead to a progressive decline in ATP production, reduced synaptic transmission efficiency, and disturbances in calcium homeostasis in neurons, thereby exacerbating the central energy deficit and further driving microglial activation, thus potentially completing the vicious cycle (179, 180).

Studies using PET provide a crucial integrative perspective in elucidating this feedback loop. Notably, a combined analysis of TSPO-PET and FDG-PET imaging indicates that neuroinflammation and metabolic dysfunction may coexist within the central nervous system, yet remain relatively independent. Some patients primarily exhibit elevated TSPO signals, indicative of an inflammation-driven phenotype, while others show reduced FDG metabolism, characteristic of a metabolic defect-driven phenotype, with no direct correlation between the two (147). Animal studies further reveal that long-term activation of glial cells may serve as the potential bridge connecting these two phenomena: activated glial cells not only release inflammatory mediators but also modulate local energy homeostasis through metabolic reprogramming (154). Systematic reviews have highlighted that the reduced activity in the insula and thalamus may represent potential clinical hubs linking peripheral inflammation, central energy deficits, and clinical manifestations (155). These collective findings support the view that PEM is not the result of a singular pathological process, but rather a dynamic network imbalance driven by the interplay of mitochondrial dysfunction, immune activation, and neuroinflammation, with distinct predominant mechanisms emerging across patient subtypes.

Crucially, all of the aforementioned central pathological changes profoundly affect the brain’s ‘interoceptive network,’ particularly the insular cortex (147, 181). The insula is a pivotal brain region responsible for perceiving bodily states (such as fatigue, pain, and energy levels) and generating corresponding emotional and motivational responses. Research has shown that in patients with chronic arthritis, functional connectivity of the insula is closely associated with subjective fatigue and pain scores, suggesting that the insula plays a central role in integrating peripheral pathological signals with subjective experience (182). Another study revealed that systemic inflammation can directly alter glucose metabolism within the insula and anterior cingulate cortex—key nodes of the interoceptive network (147). Mancini et al. further showed that decision-making behavior in chronic pain patients exhibits significantly heightened sensitivity to punishment, with posterior insular activity strongly correlated with fatigue and pain ratings, providing direct evidence of the link between reward system dysfunction and interoceptive dysregulation (183). The dysregulation of the interoceptive network mediated by neuroinflammation and central energy deficiency may be the core central mechanism by which PEM patients exhibit exaggerated perceptions of fatigue and pain after mild exertion, and this process is potentially regulated by the interaction between glial cell activation and neuronal mitochondrial dysfunction in the insular cortex. Therefore, the fatigue and cognitive impairments observed in PEM should not merely be attributed to energy depletion in muscles or neurons but should be understood as a more complex process of interoceptive dysregulation: the brain receives intense danger and low-energy signals from peripheral systems, such as muscles and the immune system, and misinterprets them as an overwhelming burden, necessitating forced rest. This amplification and misinterpretation of signals are mediated by neuroinflammation and functional changes in interoceptive centers, such as the insula.

Animal model studies provide further substantiate this mechanism, showing that inhibition of microglial activation significantly reduces the expression of IL-1β and TNF-α in the brain (184), while concurrently improving cognitive impairments and fatigue-like behaviors observed following exercise (185). Moreover, interventions aimed at enhancing mitochondrial biogenesis or promoting ROS clearance also attenuate inflammasomes activation (186, 187), suggesting that the presence of actionable interventional points within the interaction between energy metabolism and neuroimmune responses (188).

**Figure 4:**
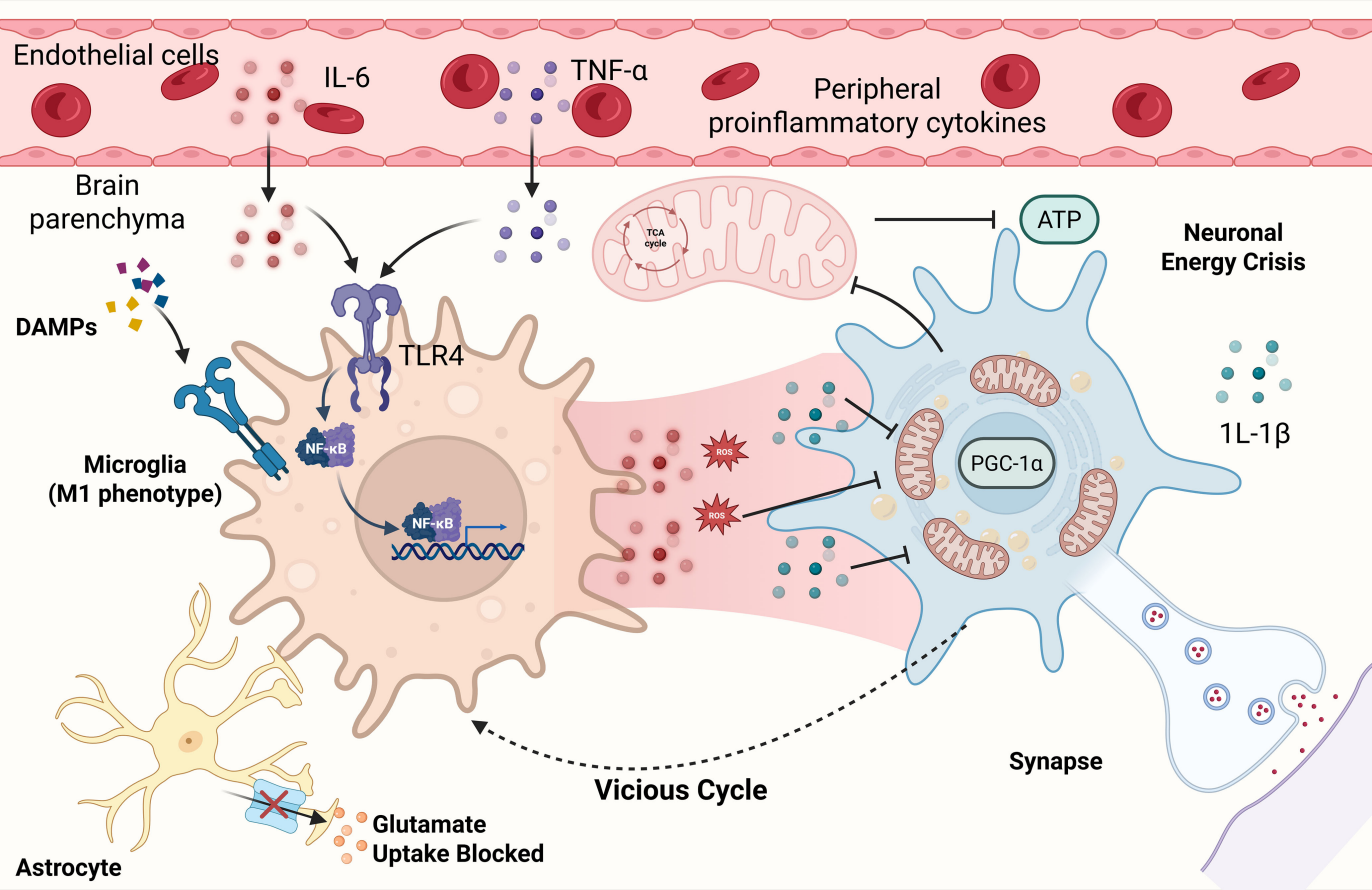
Neuroinflammation, Central Energy Deficiency, and Interoceptive Dysregulation in the Brain.

In summary, peripheral inflammatory signals may infiltrate the central nervous system via multiple pathways, eliciting aberrant activation of microglia and astrocytes, thereby underscoring the potential central role of neuroinflammation in the development of PEM. Disruption of mitochondrial energy production and diminished synaptic plasticity directly compromise brain function, heightening its vulnerability. Meanwhile, the accumulation of ROS, release of mtDNA, and activation of inflammasome establish a potential self-reinforcing cycle of energy deficiency and inflammation, which may contribute to the delayed and persistent nature of PEM symptoms. PET studies not only provide visual corroboration for this conceptual framework but also highlight the heterogeneity within patient populations, indicating that future therapeutic strategies should prioritize precise stratification to target distinct subtypes (**Figure 4**).

## 5 Discussion

The ‘metabolism-immune-neuro’ interactive framework presented in this study primarily focuses on the core pathological pathways driven by mitochondrial dysfunction. However, the clinical heterogeneity observed in PEM suggests that other pathophysiological systems, such as coagulation cascade abnormalities, autonomic dysfunction, and dysregulation of the hypothalamic-pituitary-adrenal (HPA) axis, may coexist within the same patient population and potentially interact with the mechanisms outlined in this framework. For example, endothelial dysfunction and microcirculatory perfusion abnormalities may further intensify the peripheral and central energy deficits, while autonomic dysregulation could potentially disrupt the neural regulation of energy metabolism. Future research should investigate these systems and their interactions with the ‘metabolism-immune-neuro’ axis, utilizing multi-omics stratification and longitudinal follow-up to enable precise patient stratification and the development of targeted interventions for PEM.

The pathophysiology of PEM, as described above, is not solely attributed to dysfunction in a single system. Rather, it may result from a multi-level, dynamic interaction involving energy metabolism, immune inflammation, and the nervous system. The occurrence and progression of PEM can be summarized within the following core framework.

Firstly, mitochondrial dysfunction and metabolic disturbances in energy production may represent the potential initial triggers and important underlying factors of PEM. Stressors such as physical exertion may lead to mitochondrial structural damage and functional impairment, resulting in reduced ATP synthesis, an increase in ROS production, and the accumulation of metabolic byproducts, such as lactate. This may create a potential energy crisis in both peripheral and central cells, which may directly contribute to feelings of fatigue. Furthermore, the release of DAMPs, such as mtDNA, along with metabolic signals, activates the immune system by serving as potential key triggers for immune activation. Importantly, this process reflects a transient and protective ‘toxic excitatory effect’ in healthy individuals. However, in PEM patients, underlying pathological conditions, such as persistent viral infections in PCC (25) or compromised antioxidant defenses (105), prevent the effective resolution of mitochondrial stress, thereby triggering the potential onset of a pathological cycle.

Furthermore, immune system dysregulation and inflammation may form a critical potential link between metabolic dysfunction and neurological manifestations. Mitochondrial-derived ROS and mtDNA may activate signaling pathways like the NLRP3 inflammasome, which promotes the release of pro-inflammatory cytokines, including IL-1β, IL-6, and TNF-α. This inflammatory response arises from the synergistic interplay between DAMP signals and potential PAMP signals, resulting from the persistent presence of pathogens or their components in PCC (25, 27). When activated, the immune system is highly energy-demanding. In the context of mitochondrial dysfunction, which impairs ATP production, the immune system prioritizes the sequestration and utilization of circulating glucose through metabolic reprogramming. This selfish behavior further depletes available energy substrates in peripheral tissues (such as muscle) and the central nervous system (such as the brain), thereby potentially accounting for the systemic exhaustion observed in PEM patients following even minimal exertion (189). The peripheral inflammatory can be transmitted to the central nervous system by disrupting the BBB, either through humoral or neural pathways such as the vagus nerve. Additionally, immune cell metabolic reprogramming, shifting toward glycolysis, exacerbates systemic energy depletion and potentially amplifies inflammation in peripheral and central tissues. The potential metabolism-immune-neuro interaction framework of PEM is based on multi-level molecular crosstalk: mitochondrial dysfunction generates DAMPs that activate innate immune pathways (NLRP3, NF-κB) to induce peripheral inflammation; peripheral inflammatory signals cross the BBB or via vagal nerve to activate central glial cells and initiate neuroinflammation; neuroinflammation further impairs neuronal mitochondrial function and synaptic plasticity, exacerbating central energy deficiency and interoceptive dysregulation.

Ultimately, central energy deficits and neuroinflammation together may contribute to the neurological and cognitive symptoms of PEM. Inflammatory mediators reaching the brain activate microglia and astrocytes, leading to localized neuroinflammation. The brain, a high-energy-demand organ, relies on neuronal mitochondrial function to maintain ATP production, which is crucial for synaptic transmission and plasticity (190). Mitochondrial dysfunction impairs ATP synthesis, particularly affecting the function of high-energy-demand brain regions such as the prefrontal cortex, hippocampus, and insula. In the PEM state, due to systemic metabolic dysregulation, the brain perceives an overall energy deficit. At this point, interoceptive centers like the insular cortex amplify subtle metabolic stress signals into overwhelming sensations of fatigue and pain, compelling the individual to cease activity in order to safeguard the brain’s fundamental survival needs (191). This ‘selfish’ protective inhibition may form the potential pathological foundation for the neurocognitive symptoms and delayed fatigue characteristic of PEM (147, 181).The interaction between energy depletion and neuroinflammation intensifies a potential cycle of “energy depletion - inflammation amplification”, clinically manifesting as delayed and persistent mental fatigue, cognitive dysfunction, and emotional disturbances.

In conclusion, PEM represents a susceptibility state triggered by a range of factors, including pathogens, toxins, and metabolic dysregulation, with mitochondrial stress acting as a potential primary initiator (192, 193). This process is further amplified by immune-mediated inflammation, ultimately potentially leading to central nervous system dysfunction, particularly a cascade of events that disrupts the interoceptive network. The interaction between systems creates a potential positive feedback loop through signaling molecules such as ROS, DAMPs, cytokines, and metabolites, potentially explaining the delayed onset, persistence, and multi-system nature of the symptoms. The multi-system interaction framework provides an integrated potential understanding of PEM’s complexity, suggesting that future therapeutic strategies may need to simultaneously target energy metabolism, immune regulation, and neuroprotection to effectively help disrupt this pathological cycle.
